# Linoleic acid supplementation of cell culture media influences the phospholipid and lipid profiles of human reconstructed adipose tissue

**DOI:** 10.1371/journal.pone.0224228

**Published:** 2019-10-22

**Authors:** Marie-Ève Ouellette, Jean-Christophe Bérubé, Jean-Michel Bourget, Maud Vallée, Yohan Bossé, Julie Fradette

**Affiliations:** 1 Centre de Recherche en Organogenèse Expérimentale de l'Université Laval/LOEX, Division of Regenerative Medicine, CHU de Québec -Université Laval Research Center, Québec, QC, Canada; 2 Department of Surgery, Faculty of Medicine, Université Laval, Québec, Canada; 3 Centre de Recherche de l’Institut Universitaire de Cardiologie et de Pneumologie de Québec, Université Laval, Québec, QC, Canada; 4 Department of Molecular Medicine, Faculty of Medicine, Université Laval, Québec, Canada; Fundacao Oswaldo Cruz, BRAZIL

## Abstract

Reconstructed human adipose tissues represent novel tools available to perform *in vitro* pharmaco-toxicological studies. We used adipose-derived human stromal/stem cells to reconstruct, using tissue engineering techniques, such an adipose tridimensional model. To determine to what extent the *in vitro* model is representative of its native counterpart, adipogenic differentiation, triglycerides accumulation and phospholipids profiles were analysed. Ingenuity Pathway Analysis software revealed pathways enriched with differentially-expressed genes between native and reconstructed human adipose tissues. Interestingly, genes related to fatty acid metabolism were downregulated *in vitro*, which could be explained in part by the insufficient amount of essential fatty acids provided by the fetal calf serum used for the culture. Indeed, the lipid profile of the reconstructed human adipose tissues indicated a particular lack of linoleic acid, which could interfere with physiological cell processes such as membrane trafficking, signaling and inflammatory responses. Supplementation in the culture medium was able to influence the lipid profile of the reconstructed human adipose tissues. This study demonstrates the possibility to directly modulate the phospholipid profile of reconstructed human adipose tissues. This reinforces its use as a relevant physiological or pathological model for further pharmacological and metabolic studies of human adipose tissue functions.

## Introduction

Adipose tissue (AT) is now recognized as a key endocrine organ with dramatic influence on general physiology and key involvement in cardiovascular diseases. The composition of AT is primarily influenced by alimentary intakes and its function is dependent on its composition. One of the principal molecules found in AT are lipids, which actively regulate metabolic processes both inside adipocytes (triglycerides, energy storage) and in cell membranes (glycerophospholipids, signaling) [1]. Indeed, polyunsaturated fatty acids (PUFAs) influence vital aspects of the cellular physiology, including membrane fluidity [2], metabolism [3–5], protein folding and functionality [6], lipid rafts [7, 8], signaling [9, 10], trafficking processes [11, 12] and inflammation [13]. While saturated fatty acids (FAs) can be produced by mammalian cells from sugar (lipogenesis), PUFAs need to be provided from the diet. The endocrine action of AT is mediated by secretion of hormones including adipokines, that act directly on key tissues and organs, including the nervous system, to regulate adiposity, glucose homeostasis, food intake, blood pressure, inflammation, lipid metabolism and angiogenesis [14, 15].

Adipocytes within the adipose depots are specialized cells that possess the ability to incorporate FAs from their surrounding environment to either produce triglycerides (TG), be incorporated into cell-membrane phospholipids or activate transcription factors associated with adipocyte differentiation [16]. Most of the current knowledge on adipocyte functions has been gained either from animal models or monolayer cell cultures *in vitro*. For example, one of the main adipocyte model is based on 3T3-L1 cells, a murine immortalized cell line [17–21]. Classical cell culture-based models recreate only partially the complex three-dimensional (3D) human tissue architecture. It is possible to reconstruct adipose tissue by tissue engineering in a highly controlled environment. Such human reconstructed adipose tissues (hrAT) are interesting novel tools suitable for pharmaco-toxicolocal studies that could favorably replace the use of immortalized cell lines, monocultures or animal cells [22–24]. Indeed, these 3D models are particularly suited to study adipose tissue formation, maturation and remodelling *in vitro*, as well as the tissue-specific gene expression [24]. It is possible to use human stromal/stem cells extracted from native adipose tissue to reconstruct such a 3D model using a self-assembly approach of tissue engineering [25]. Using this engineering approach [26], the *in vitro*-produced extracellular matrix is secreted, deposited and organized by mesenchymal cells into a cohesive structure exempt of exogenous material, forming a tissue featuring a physiological extracellular matrix environment. Our hrAT model has been previously characterized demonstrating adipose tissue characteristics such as lipid droplet accumulation and production of leptin, PAI-1 and angiopoietin-1 proteins [23]. They also shown alpha adrenergic stimulated lipolytic activity and secretion of adipokines including leptin and adiponectin [25]. It has also been used to characterize the impact of inflammatory mediators such as tissue necrosis factor-α [22, 23]. While mRNA and protein expression of the main adipokines and proangiogenic factors expressed by hrAT has been characterized previously [23], however, this model has not been characterized yet in terms of global transcriptomic and fatty acid profiles in comparison with native adipose tissue.

Omega (ω) 3 and ω6 fatty acids are key molecules influencing the inflammatory response. Linoleic (LA, ω6, 9c12c-18:2) and α-linolenic (αLA, ω-3, 9c12c15c-18:3) acids are called essential fatty acids because they need to be provided from the diet [27, 28]. Fatty acids are the constituent of the hydrophobic tail of glycerophospholipids that form the cell membrane. Incorporation of PUFAs increases the lateral movement of transmembrane proteins [29, 30]. PUFAs are also essential precursors of the eicosanoid family of hormones [31]. Linoleic acid is present in many plant seed oils. It is the indirect precursor of arachidonic acid that can be converted to various eicosanoids in mammals. This process is dependent on the activity of the delta-6-desaturase (Δ6-desaturase) [32]. This enzyme can be blocked by using a specific inhibitor (SC-26196) to prevent the conversion of LA in arachidonic acid and downstream synthesis of eicosanoids.

Since food intake directly influences the lipid profile of the adipocytes, the same process is likely happening when cells are expanded *in vitro*. The culture medium aims to mimic the natural environment of tissues. Foetal calf serum (FCS) is commonly used to provide nutrients and growth factors to cells *in vitro*. Composition of FCS is inconsistent between batches and those variations have important impact on cell culture. Indeed, variations in serum were shown to influence the fatty acid composition of established cell lines [33].

In this study, we engineered human 3D adipose tissues *in vitro* to compare the differences in adipogenic differentiation, triglycerides accumulation and phospholipids profiles between these reconstructed tissues and native AT using transcriptomic and analysis of fatty acids. Our hypothesis is that the lipid composition of the engineered adipose tissues will reflect their FA intakes and therefore will be influenced by the fatty acid composition of the culture medium and by a supplementation with PUFAs. Our results unveil differences in lipid profiles that could be restored by providing essential fatty acids to cells, demonstrating the versatility of the model as a tool to investigate human adipocyte’s metabolic responses.

## Materials and methods

### Ethic statements

All protocols involving human tissues and cells in culture followed the tenets of the declaration of Helsinki and were approved by the institutional Ethics Committee of the CHU de Québec–Université Laval (#DR-002-1117). Written informed consent was obtained from the patients for the use of their tissues and cells for research.

### Cell culture

**Fig 1:**
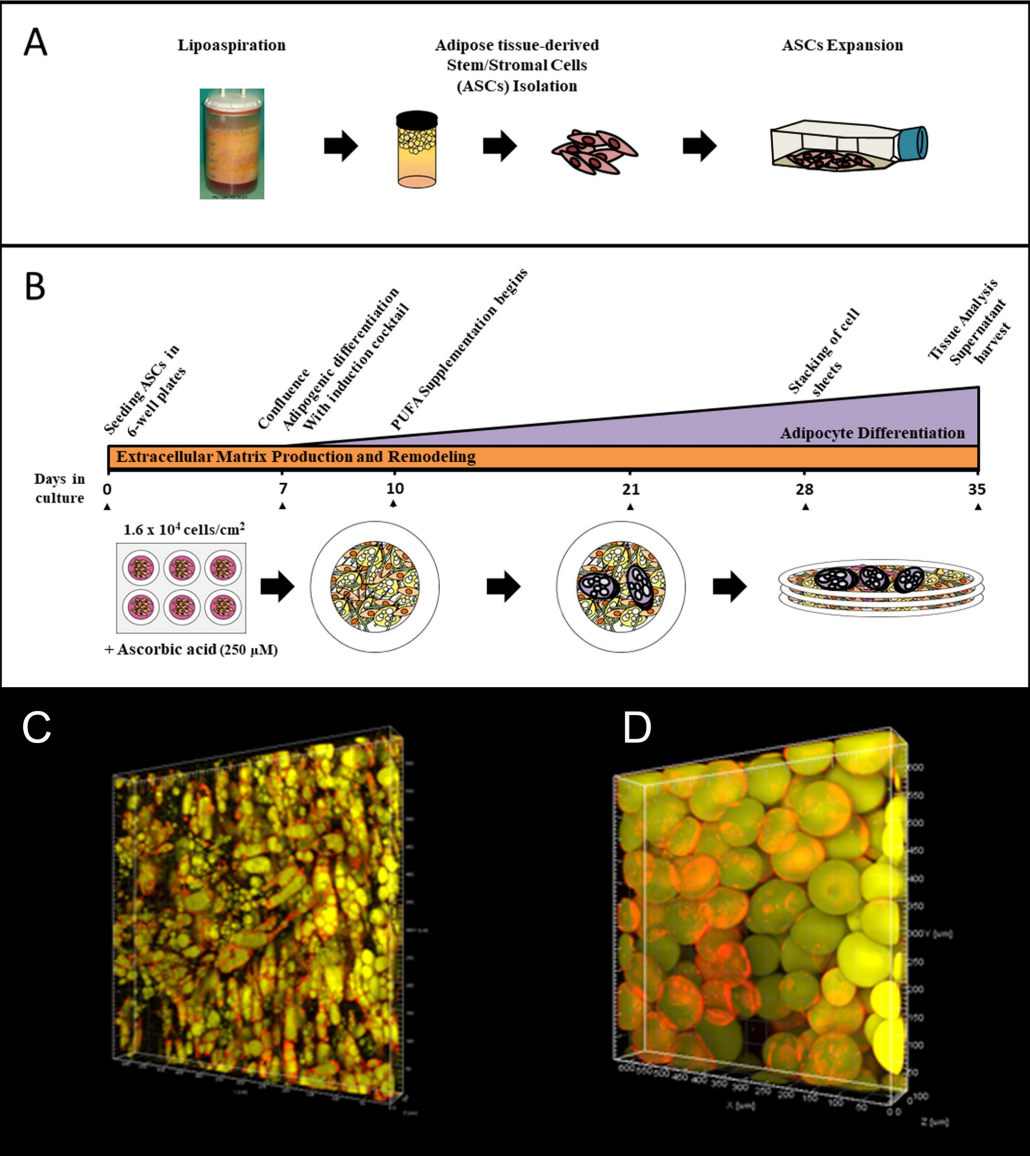
Production and imaging of reconstructed human adipose tissues. (A) Adipose-derived mesenchymal stem/stromal cells (ASCs) were isolated from lipoaspirated fat obtained from healthy donors, and expanded in culture. (B) ASCs were plated in 6-well plates with ascorbic acid to stimulate matrix production/organization. Adipogenic differentiation was induced at day 7 by addition of the induction cocktail. Three days later, the medium was substituted for adipocyte maintenance medium. For tissue supplemented with linoleic acid, the supplementation began at day 10. On day 28, three cell sheets were superimposed to form thicker constructs. Control connective tissue sheets were produced by omission of the induction cocktail and the adipocyte medium was replaced by 10% FCS expansion medium. Analyses were performed at day 35). Confocal imaging of (C) reconstructed human adipose tissue and (D) native resected fat. Perilipin expression is shown in red while Nile Red-stained adipocytes appear in yellow.

Subcutaneous human adipose tissue was obtained and processed as described previously [25], from non-obese women undergoing cosmetic surgery procedures (N = 4; age 30–54, mean age 40; body mass index (BMI) 21–25 kg/m^2^, mean BMI 23 kg/m^2^). Subcutaneous AT (native AT samples) were harvested with a 4 mm canulae, rinsed, flash-frozen in liquid nitrogen and preserved at -80°C prior to RNA extraction. The viable adipose-derived stem/stromal cells (ASCs), obtained after extraction, were counted using trypan blue exclusion assay and seeded at a density of 8 x 10^5^ cells/cm^2^ for *in vitro* expansion and batch cryopreservation after primary culture (P0) (Fig 1A). Experiments were performed on thawed cells at passage (P) 2 to 5. Cells were seeded at a density of 1.5 x 10^4^ cells/cm^2^ and cultured in expansion medium consisting of 1:1 DMEM: Ham’s F12 medium (DMEM-F12) (Invitrogen, Oakville, Ont., Canada) supplemented with 10% FCS (HyClone, Logan, UT) and antibiotics (100 U/ml Penicillin and 25 mg/ml Gentamicin; Sigma-Aldrich, Oakville, Ont., Canada). Culture medium was changed three times a week (37°C, 95% relative humidity, 8% CO_2_) and passaged weekly. For the analysis of the fatty acid composition of the different lots of FCS, expansion medium were supplemented with 10% serum from five different lots.

### Production of reconstructed human adipose tissues

The ASCs were seeded at a density of 1.6 x 10^4^ cells/cm^2^ in 6-well plates and expanded until confluence. For the entire duration of the experiments, the cultures were grown in presence of a wave-like motion (dynamic condition [34, 35]) and culture medium was supplemented with 50 μg/ml of ascorbic acid (vitamin C) to favour matrix accumulation [25]. In order to produce adipocyte-containing tissue sheets, the ASCs were differentiated into adipocytes (7 days after seeding) by supplementing the DMEM-F12 medium with an adipogenic cocktail containing 100 nM insulin (Sigma-Aldrich), 0.2 nM T3 (Sigma-Aldrich), 1 μM dexamethasone (Sigma-Aldrich), 1 μM rosiglitazone (Cayman chemical, Cedarlane, Hornby, Ont., Canada) and 25 μM 3-isobutyl-1-methylxanthine (IBMX; Sigma-Aldrich). After 3 days of induction of differentiation, this induction medium was substituted for the adipocyte maintenance medium (expansion medium supplemented with 100 nM insulin, 0.2 nM T3 and 1 mM dexamethasone). Non-induced controls were cultured in the expansion medium and termed human reconstructed connective tissues (hrCT). After 28 days, tissue sheets of the same category (3.5 cm^2^ surface area) were stacked three by three in order to produce thicker tissues. Those three-layered adipose and control tissues were cultured for an additional week in presence of ascorbic acid in order to allow fusion between tissue sheets before analysis [36].

For supplementation assays, three days after the start of the induction process (day 10), and until tissues were analysed at day 35, 150 μM of linoleic acid (L1376; Sigma-Aldrich), 100 nM Δ6 desaturase inhibitor (SC-26196; Sigma-Aldrich) or their corresponding vehicles (ethanol 0.1% and/or DMSO 0.5% V/V) were added directly to the cell culture well at each medium change, when indicated (Fig 1B). Linoleic acid was diluted in 100% ethanol while the inhibitor SC-26196 was diluted in pure DMSO.

For gas chromatography, effect of inhibitor and LA supplementation assays, samples from 4 different donors were analysed, each experiment was performed in triplicates.

### RNA extraction

Total RNA was isolated from 1 x 10^6^ cultured cells using the RNeasy Mini Kit and Qiashredder (Qiagen, Mississauga, Ont., Canada). Reconstructed and native adipose tissues were flash-frozen in liquid nitrogen and preserved at -80°C. Tissues were homogenized with a TissueRuptor (Qiagen) and total RNA was isolated using the RNeasy Lipid tissue Mini Kit (Qiagen). The RNA extraction procedures included a DNase I treatment to remove genomic DNA. The extracted RNA was eluted in sterile RNAse free water and quantified using a NanoDrop ND-1000 Spectrophotometer (NanoDrop Technologies, Wilmington, DE); the concentration was adjusted to 1 μg/μl.

### Microarray analysis

Microarray experiments were performed at the McGill University and Génome Québec Innovation Centre, Montréal, Qc, Canada. Total RNA (250 ng) from hrAT (N = 4), hrCT (N = 4) and native subcutaneous adipose tissues (N = 4) was amplified with the Illumina TotalPrep^™^ RNA Amplification Kit according to the manufacturer’s instructions (Ambion, Austin, TX). Hybridizations were performed on the Illumina Human Whole-Genome-6 v3 (Illumina, San Diego, CA) according to manufacturer’s instructions, using 1.5 μg of cRNA. Signals were developed with streptavidin-Cy3 and the BeadChip were scanned with the Illumina BeadArray Reader.

The raw data were log2-transformed and quantile normalized with the *lumi* package in R [37, 38]. All probes were considered in the analysis and no filter was applied to eliminate genes with low expression signals. The Significance Analysis of Microarrays (SAM) method [39] was utilized to identify probes differentially expressed between hrAT and native human fat. The false discovery rate (FDR) and the fold change (FC) threshold for significance were set at 5% and 2.0, respectively. Each probe was treated independently and transcripts interrogated by multiple probes were not summarized.

Gene expression data are available in NCBIs Gene Expression Omnibus (GEO, http://www.ncbi.nlm.nih.gov/geo/query/acc.cgi?acc=GSE123685) through GEO Series accession number (GSE123685).

Biological pathway analyses were performed with the Ingenuity Pathway Analysis (IPA) web-based software (Ingenuity® Systems, www.ingenuity.com). The significance value associated with Functional Analysis for a dataset is a measure of the likelihood that the association between a set of Functional Analysis genes in your experiment and a given process or pathway is due to chance. The p-value associated with a biological process or pathway annotation is a measure of its statistical significance with respect to the Functions/Pathways/Lists Eligible molecules for the dataset and a Reference Set of molecules. The p-value is calculated with the right-tailed Fisher's Exact Test. The Molecule Activity Predictor (MAP) tool implemented in IPA was also utilized to predict the upstream and/or downstream effects of activation or inhibition of molecules in pathways given the set of neighbouring genes with measured expression. MAP enables the visualization of the overall effect on a pathway.

### Histology

Reconstructed adipose tissues were fixed using 3.7% formalin (VWR, Montreal, QC, Canada) and embedded in paraffin. Sections of five micrometers were cut and stained with Masson’s trichrome [40, 41] using aniline blue, Weigert’s haematoxylin and Ponceau-Fuchsin-stains.

### Staining of tissues (perilipin and Nile red) for confocal imaging

Free-floating formalin-fixed native and reconstructed adipose tissues were washed in 1% W/V bovine serum albumin (Sigma-Aldrich) in PBS [23, 42]. Tissues were incubated at 4°C for 48 hours with a rabbit anti-human perilipin primary antibody (Cell Signaling Technology, Beverly, MA). Secondary antibody was Alexa-594 conjugated goat anti-rabbit IgG (Thermo Fisher Scientific, San Jose, CA). To visualize the lipid content, samples were incubated for a minimum of 2 hours in 200 ng/ml Nile Red solution (N-1142 (0151–12), Life technologies, Burlington, Ont., Canada). Images were acquired using a Zeiss LSM700 scanning laser confocal microscope apparatus and the Zen software (Carl Zeiss MicroImaging, Toronto, Ont., Canada).

### Gas chromatography analysis

Approximately 50 mg of hrAT cultured under various types of supplementations were homogenized using a glass potter. Lipids were extracted along with C-15 phosphatidyl choline, as internal standard (Avanti Polar Lipids, Alabaster, AL), in a chloroform:methanol (C-M) mixture (2:1, V:V) according to a modified Folch method [43]. Half of the extraction of lipids was kept for total tissue FAs analysis and the other half was used to isolate the phospholipid fraction by thin layer chromatography with isopropyl ether:acetic acid (96:4, V:V) as the developing solvent. FAs of the total lipid and phospholipid fractions were then methylated using methanol:benzene (4:1, V:V) and acetyl chloride as previously published [44, 45].

The FA profiles were obtained by capillary gas chromatography using a temperature gradient on a HP5890 gas chromatograph (Hewlett Packard, Toronto, Ont., Canada) equipped with a HP-88 capillary column (100 m x 0.25 mm i.d. x 0.20 μm film thickness; Agilent Technologies) coupled with a flame ionization detector (FID). Helium was used as carrier gas (split ratio 1:40). FAs were identified according to their retention time, using a standard mixture of 42 FAs as a basis for comparison (FAME 37 mix, Supelco Inc., Bellefonte, PA), the C15:0 FA, as well as the following FAs: C22:5 ω6 (Larodan AB, Malmo, Sweden), C22:5 ω3 (Supelco Inc.), C22:5 ω3 cis-12 and a mixture of 31 FAs (GLC-411; NuChek Prep Inc, Elysian, MN). Finally, a mixture of trans FAs containing: C18:2 ω6 cis/trans (Supelco Inc), a mixture of C18:3 ω3 cis/trans (Supelco Inc.), and C14:1 trans-9, C16:1 trans-9 and isoforms of C18:1 (cis-6, cis-11, cis-12, cis-13, trans-6, trans-11) (Supelco Inc.) were also used as standards. Results are expressed as percent of total FAs and as milligrams of FAs per gram of tissue.

### Statistical analysis

The assumptions of multivariate normality and homogeneity of covariance matrices were not met for all 42 FAs analysed for the six cultures conditions; thus, a robust multivariance analysis of variance (MANOVA) on the ranked data was performed using Munzel and Bruner’ method (2000) implemented in R using the “mulrank()” function [46].

The robust MANOVA was followed by 42 Bootstrapped 1-way ANOVA (N = 1000, Bias corrected). A Benjamini-Hochberg correction for the control of the false discovery rate (5%) was applied to the bootstrapped ANOVA test-results to determine which FAs were significantly different between the six conditions. The effect size, based on variance for 1-way ANOVA omnibus test, was calculated for FAs after a holm/Bonferroni correction. Results are presented as mean ± standard deviation (SD).

## Results

### The transcriptome of reconstructed human adipose tissue shows differentially expressed genes (DEGs) compared to native fat

The self-assembly approach of tissue engineering allows the production of hrAT featuring numerous adipocytes and abundant endogenous extracellular matrix components. Confocal imaging was performed to compare adipocyte morphology of the hrAT and the native fat (Fig 1C and 1D). Intracellular lipid droplets stained by Nile red (yellow), showed that adipocytes from hrAT were composed of multilocular lipid droplets after 28 days of differentiation (Fig 1C). In contrast, human native adipose tissues (mean age 40 years old) were composed of adipocytes containing a unilocular lipid droplet (Fig 1D). Expression of lipid droplet-associated protein (PLIN) or perilipin (shown in red), an essential protein that protects lipids from lipase degradation, was detected around the adipocyte’s lipid droplet in both hrAT and native fat samples.

**Fig 2:**
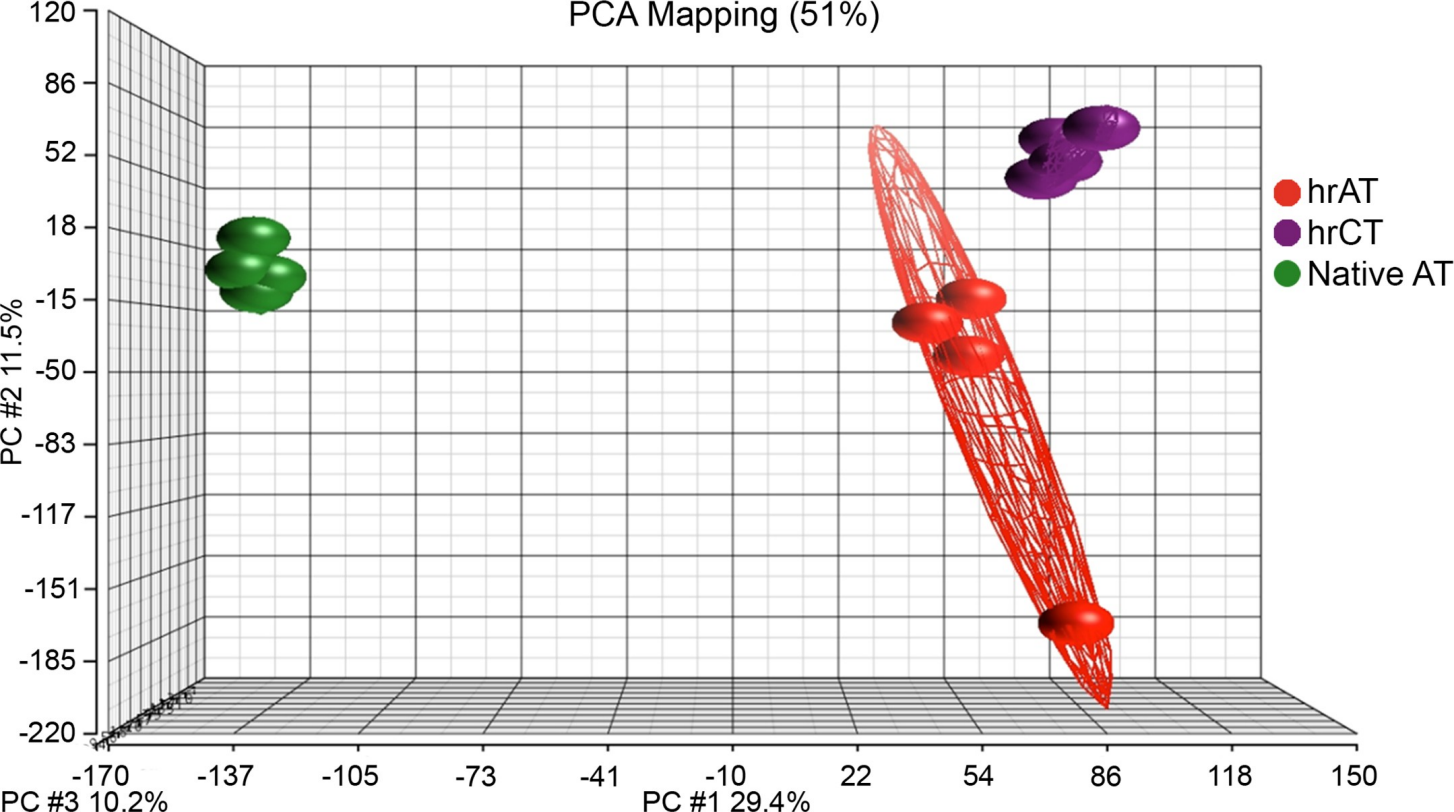
Principal component analysis (PCA) of the transcriptomes. PCA reveals the variance in human reconstructed connective (hrCT; Purple, N = 4) and adipose (hrAT; Red, N = 4) tissues reconstructed using adipose stromal/stem cells compared to native subcutaneous AT (Green, N = 4). mRNA expression levels (37 805 transcripts) were reduced to three principal components visualizing 51% of the variance in the data. Each dot represents a distinct sample and the ellipses around the dots illustrate two times the standard deviation.

Bioinformatic analysis of the transcriptome revealed that 8% of transcripts were differentially expressed between hrAT and native fat (2 915 differentially expressed genes (DEGs) with FDR < 5% and FC > 2). Principal component analysis (PCA) was performed to visualize the transcriptome (37 805 mRNA transcripts) and to highlight the differences between samples/conditions. The gene expression profile of adipose tissue donors clusters tightly on the PCA plot (Fig 2). The hrAT display more variability among cell populations compared to non-induced reconstructed connective tissues (hrCT), suggesting heterogeneity in response to the induction cocktail.

### Ingenuity pathway analysis revealed enriched pathways in engineered compared to native adipose tissue

**Fig 3:**
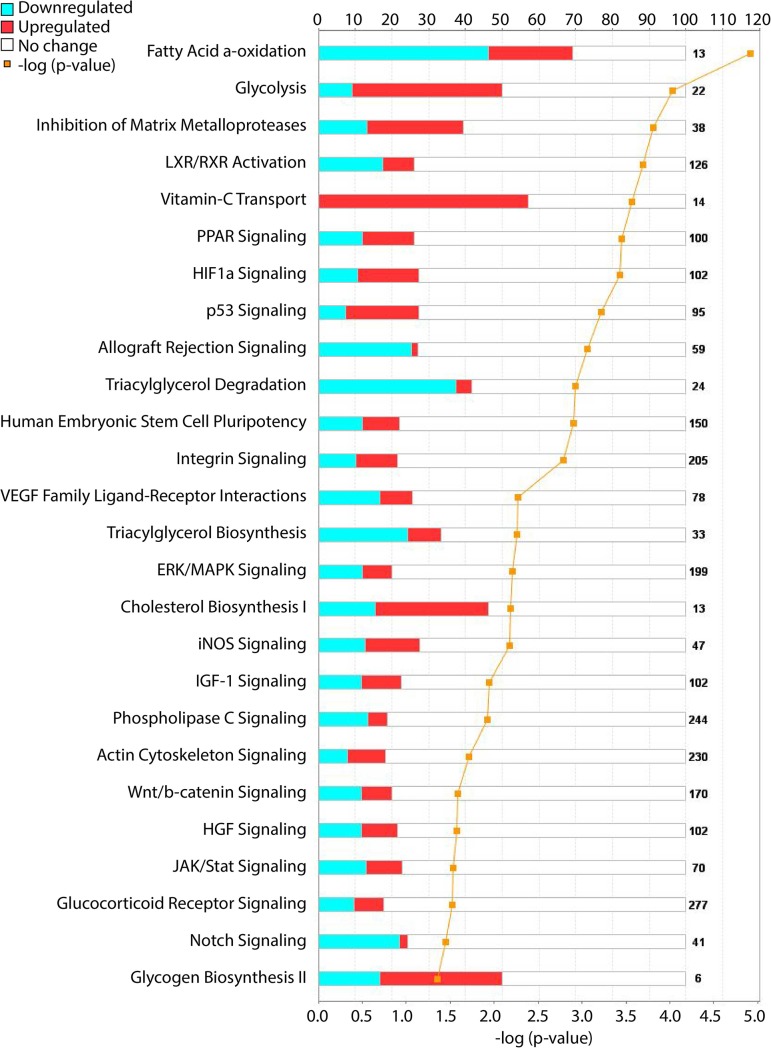
Selections of canonical pathways enriched for DEGs associated with adipocyte’s functionality. Significantly up- or downregulated genes (p<0.05 and 2-fold change) in engineered compared to native adipose tissues were analysed with IPA. Bars represent the percentage of genes significantly altered in the pathway. The numbers of genes included in the pathway are indicated at the right. Negative log of the p-values are represented by the yellow line. Downregulated and upregulated genes in each pathway are represented in blue and red, respectively.

Pathways enriched for DEGs were identified in engineered (hrAT) compared to native adipose tissues (Fig 3). As expected, since the hrAT culture media was supplemented with vitamin C (self-assembly approach of tissue engineering), genes included in the vitamin C transport pathway were upregulated when compared to native adipose tissue. Other extracellular matrix-associated pathways such as integrin signaling, actin cytoskeleton signaling, metalloprotease signaling were also enriched for DEGs. Main secretome-associated pathways such as vascular endothelial growth factor (VEGF), insulin growth factor-1 (IGF-1) signaling as well as differentiation pathways such as liver X receptor/Retinoid X receptor (LXR/RXR) and peroxisome proliferator-activated receptor (PPAR) signaling showed less than 25% change in gene expression compared to native fat. In contrast, 40% of genes associated with the glycolysis I signaling pathway were upregulated when compared to native adipose tissue. The highest number of downregulated genes in hrAT was related to pathways associated with fatty acid α-oxidation, triacylglycerol synthesis and degradation, as well as phospholipase C signaling (Fig 3).

### Differences in transcriptome of human reconstructed and native adipose tissues influence biological function

Using the MAP tool implemented in IPA, we evaluated whether variations observed in specific pathways associated with engineered adipose tissue affect biological functions. Table 1 enumerates significant biofunctions and the direction of the effect observed in reconstructed compared to native AT. Higher activation state of biofunctions related to cell viability, proliferation and cell survival is coherent with the attributes of a tissue developing and expanding *in vitro*. More surprisingly, these *in silico* analyses predicted lower activation state for biofunctions related to the synthesis of triacylglycerols and hydrolysis of lipids.

**Table 1:** Predicted activation state of enriched biofunctions modulated in reconstructed human adipose tissues compared to native fat.

Category	Function	p-Value	Predicted Activation State	Regulation Z-score
Proliferation	Proliferation of cells	5.76E-18	Increased	2.263
Adhesion	Adhesion of connective tissue cells	8.93E-08	Increased	2.546
Tumorigenesis	Tumorigenesis	8.54E-51	Decreased	-0.149
Survival	Cell survival	1.49E-06	Increased	2.513
Cell viability	Cell viability	6.73E-05	Increased	2.337
Differentiation	Differentiation of cells	2.65E-04	Decreased	-3.191
Synthesis	Synthesis of triacylglycerols	9.49E-04	Decreased	-2.584
Hydrolysis	Hydrolysis of lipids	2.91E-03	Decreased	-2.573

The lipid metabolism biofunctions (synthesis and hydrolysis) was further subdivided to refine putative pathways that mediate their predicted lower activation state in adipocytes. Fourteen to 27% of genes in pathways involved in lipid metabolism biofunctions were downregulated (Table 2) including genes coding for many classes of phospholipases: (fold change; number of pathways) PLA2G7 (-2.02; 11); PNPLA2 (-5.50; 22); PLCL2 (-2.02; 1); PLD1 (-2.39; 25) and apolipoproteins: APOL3 (2.02; 21), ALOX5 (-3.02; 1).

**Table 2:** Pathways included into lipid metabolism biofunctions.

Pathways	Downregulated genes/ Total of genes in the Pathway	
Linoleic acid	53/261	20%
α-linoleic acid	23/110	21%
Arachidonic acid	17/121	14%
Fatty acid metabolism	29/127	27%
Fatty acid elongation	47/237	20%
Fatty acid biosynthesis	38/126	23%
Biosynthesis of unsaturated fatty acids	53/261	20%

### The low level of linoleic acid in culture medium translates into a deficiency in this essential fatty acid in reconstructed adipose tissues

**Fig 4:**
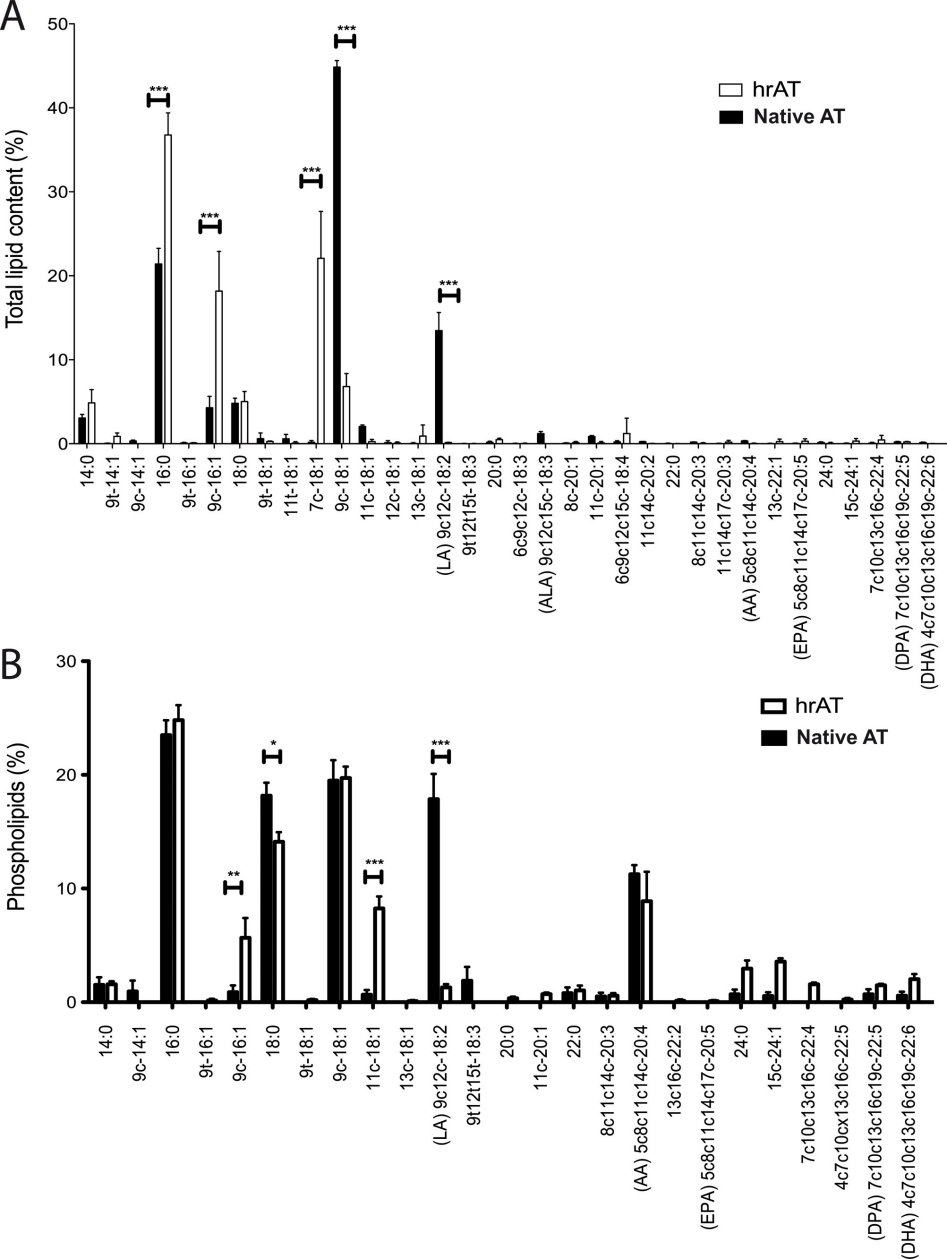
**(A) Total lipid and (B) phospholipid profiles of reconstructed human adipose tissues compared to native adipose tissue.** Lipids were extracted by chloroform/methanol and analysed by gas chromatography with a temperature gradient and helium as the mobile phase with a flame ionization detector. (N = 4, * = p < 0.05; ** = p < 0.01; *** = p < 0.001).

To verify the prediction of a low level of arachidonic acid and downstream lipoproteins, we performed a quantitative analysis of the lipid content in hrAT compared to native fat by gas chromatography and reported the proportion of each FA overall (total lipid content, Fig 4A) and in the membrane (phospholipids, Fig 4B). Some lipids were significantly modulated (*16*:*0*, *18*:*0*, *9c-16*:*1*, *7c-18*:*1*, *9c-18*:*1*, *11c-18*:*1*, *9c-12c-18*:*2*), as shown on either one or both graphs. Interestingly, the proportion of oleic acid (9c-18:1; total lipid content) was lower in hrAT than in native fat. In contrast, its naturally occurring isomer, petroselinum acid (7c-18:1), was highly abundant in hrAT as compared to its low level in native tissue (Fig 4A). Moreover, this analysis revealed that LA, the precursor of arachidonic acid, represents a significantly lower proportion of the total lipid content (Fig 4A) as well as total phospholipid content (Fig 4B) in the hrAT compared to native fat. Quantitative values (total lipids) of LA in hrAT and native AT were respectively 0.4 ± 0.2 mg FAs/g of tissue (0.9% of total FAs) and 52.0 ± 12.0 mg FAs/g of tissue (13% of total FAs).

**Fig 5:**
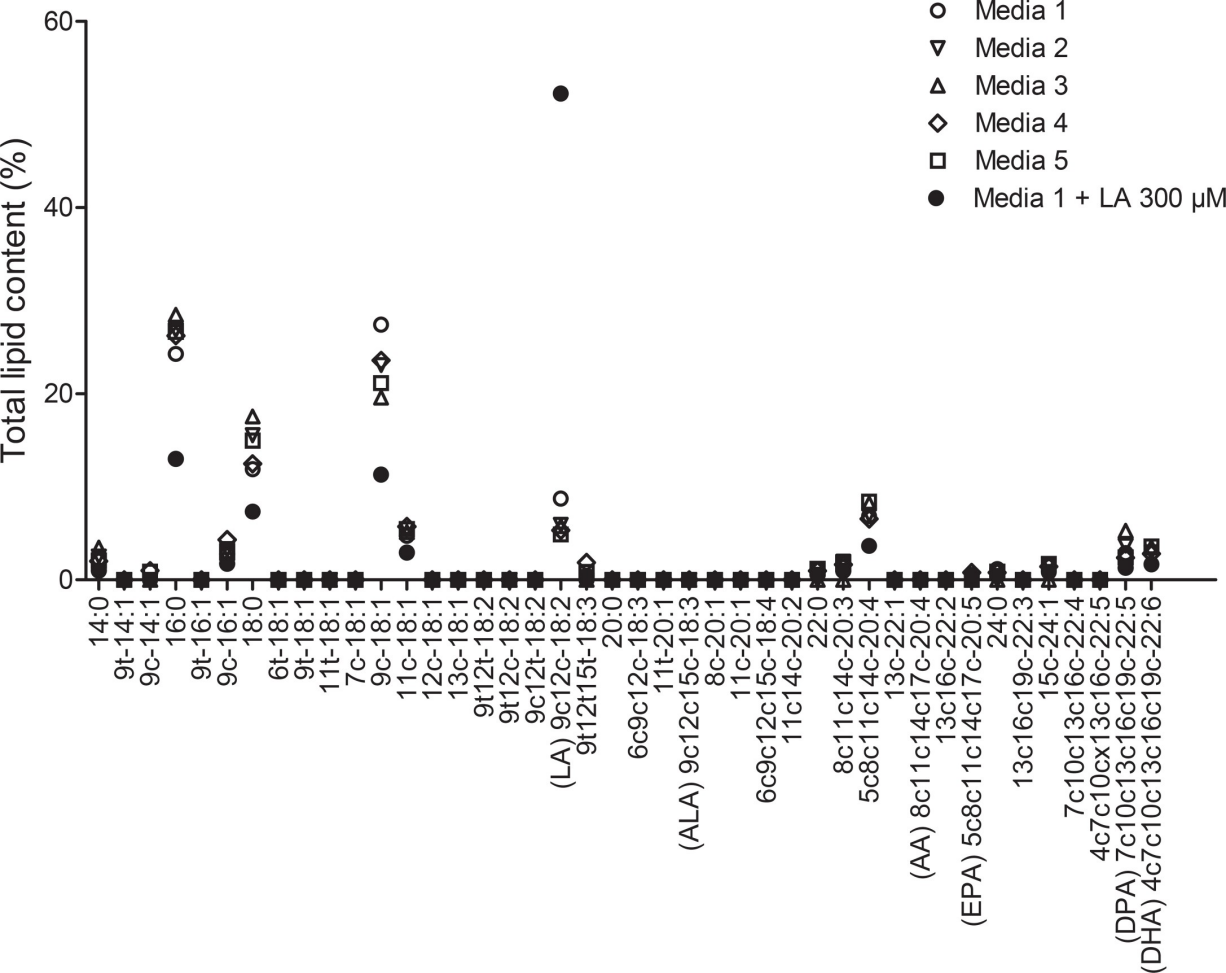
Evaluation of the lipid content in serum-supplemented culture media used for tissue reconstruction. Media 1 to 5 differ by the FCS lot used. Media + 300 μM LA (9c12c-18:2) contains the same FCS as media 1. Absolute mean value of 9c12c-18:2 in the 5 unsupplemented media was 2.13 μg/ml. A concentration of 56.0 μg/ml was detected in media 1 supplemented with linoleic acid at a concentration of 84.1 μg/ml. This media was used for tissue reconstruction with supplementation at the optimal concentration for culture (150 μM).

The fatty acid composition of the culture media containing 10% of FCS from different lots used for hrAT reconstruction was then evaluated by gas chromatography (Fig 5). This analysis revealed that only traces of LA (2.1 ± 2.3 μg/ml, mean N = 5) were present in the five media tested (without cells). Supplementation of the media with 300 μM (84.1 μg/ml) of linoleic acid increases the proportion of this fatty acid and a concentration of 56.0 ± 24.4 μg/ml was measured. The mean fatty acid level in the cell culture media used for tissue reconstruction was 46.3 ± 49.4 μg/ml.

### Supplementation of culture medium with LA modifies the fatty acid profile of reconstructed human adipose tissue

**Fig 6:**
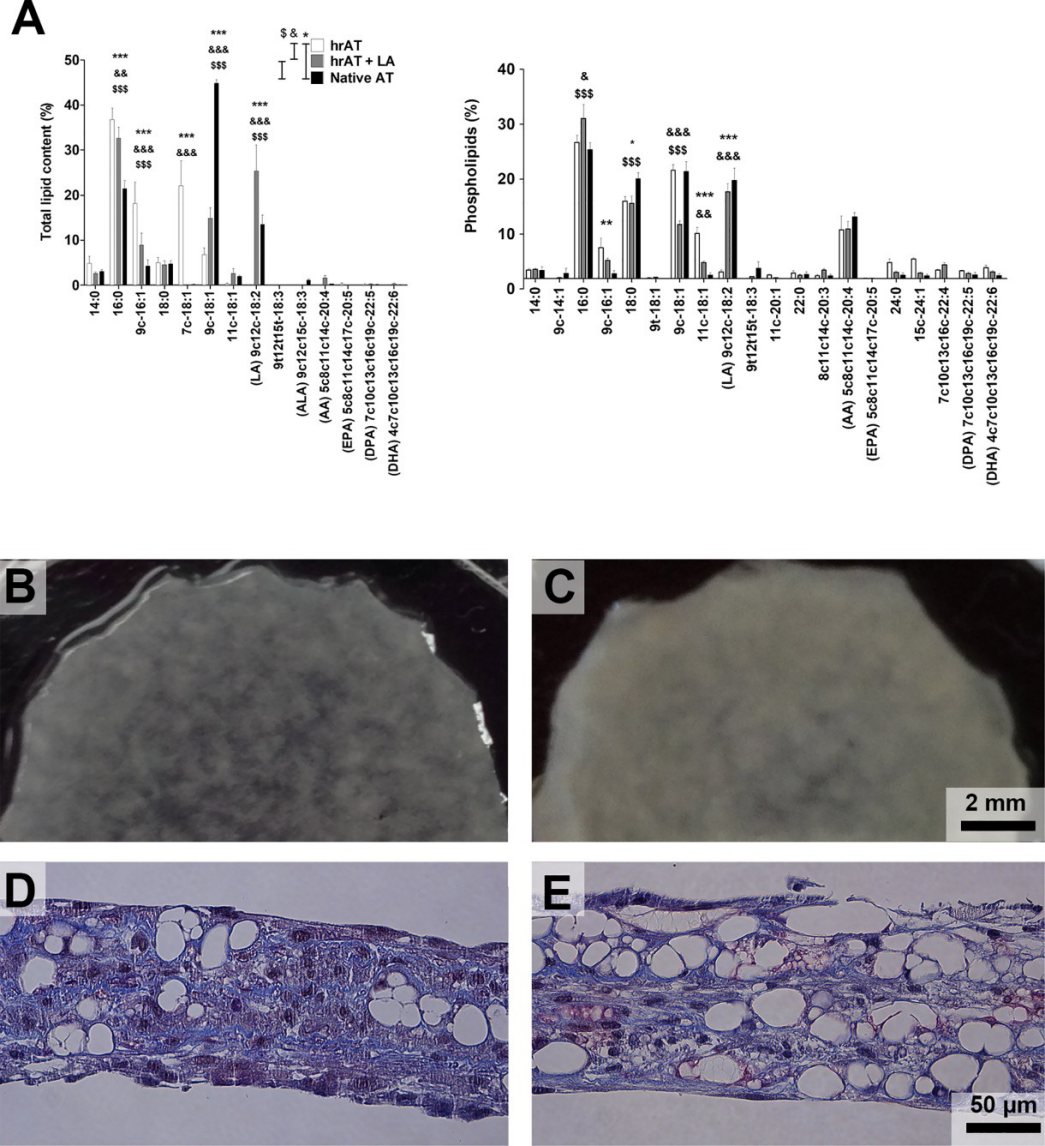
Effect of LA supplementation on lipid accumulation in engineered adipose tissues. (A) Analysis of the total lipid and phospholipid profiles by gas chromatography of human reconstructed adipose tissues (hrAT) supplemented with 150 μM LA during 25 days compared to native fat and to the control tissues cultured with the vehicle only (ethanol). Fatty acids with a value lower than 1% for each condition in total lipid content are not shown (n = 5). (B-C) Macroscopic images of reconstructed adipose tissues cultured in (B) control culture media or (C) in media supplemented with LA. (D-E) Masson’s trichrome staining of transversal sections of reconstructed adipose tissues cultured in (D) control culture medium or in (E) LA-supplemented medium. * = between hrAT and native AT; & = between hrAT and hrAT+ LA; $ = between hrAT + LA and native AT, * = p < 0.05; ** = p < 0.01; *** = p < 0.001.

We evaluated if supplementation of the culture medium with LA could influence the fatty acid profile of the hrAT and impact the differentiation and maturation of the adipocytes. Gas chromatography analyses revealed that the addition of 150 μM of LA to the culture medium efficiently modified the relative abundance of LA in both the total lipid and in the phospholipid fraction (Fig 6A). Interestingly, the oleic acid isomer petroselinum acid (7c-18:1) was reduced to native AT level when supplemented with LA (Fig 6A). Visually, macroscopic observations (Fig 6B and 6C) and histological staining of transverse tissue sections of hrAT (Fig 6D and 6E) indicated that the adipose tissue model, with and without LA supplementation during tissue production, lead to opaque adipose tissue (Fig 6C) that featured numerous adipocytes embedded in the matrix (Fig 6E).

**Fig 7:**
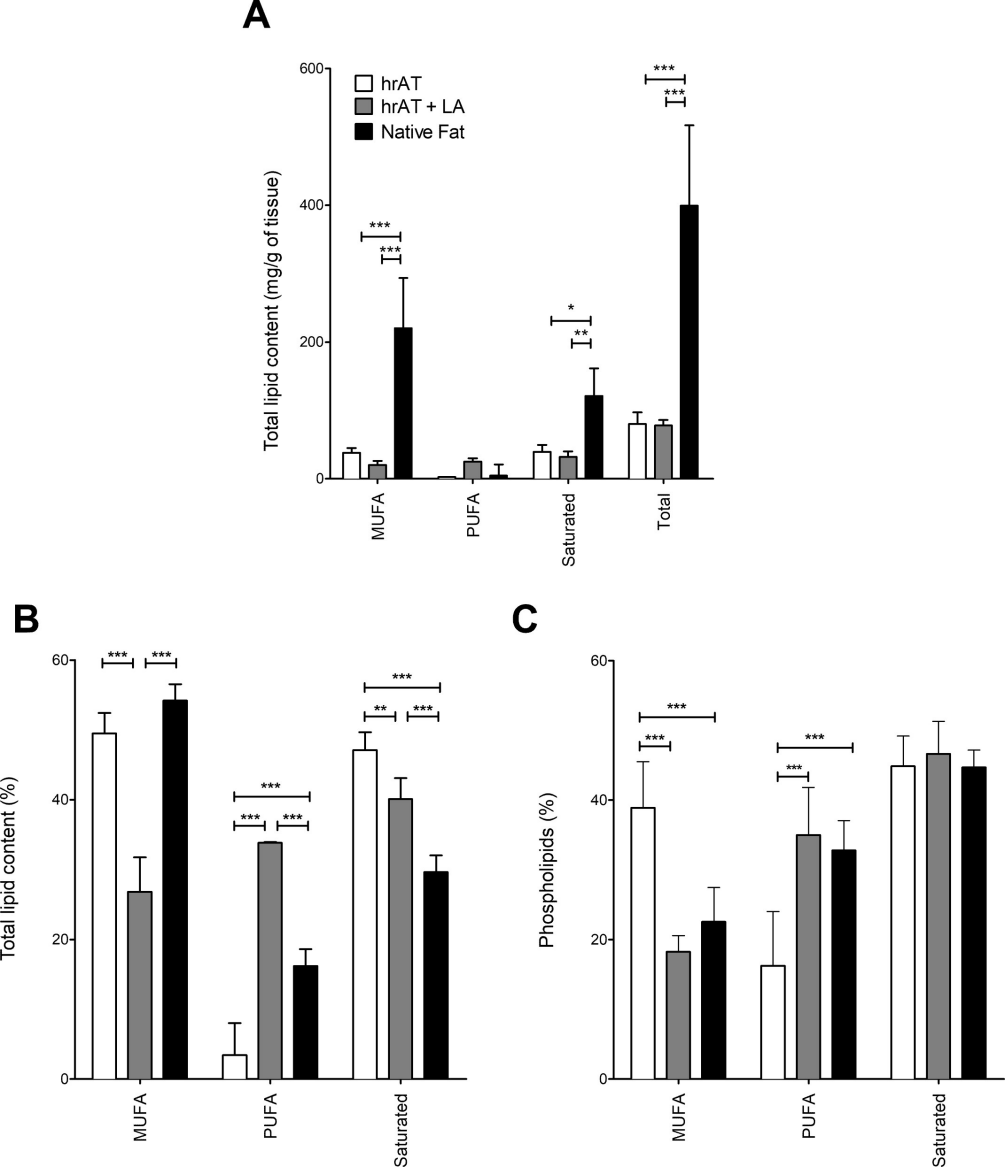
**(A-B) Total lipid and (C) phospholipid profiles of human reconstructed adipose tissues compared to native adipose tissue.** Lipids were extracted by chloroform/methanol and analysed by gas chromatography with a temperature gradient and helium as the mobile phase with a flame ionization detector. Results were clustered by unsaturation level of fatty acids (FAs). N = 4, * = p < 0.05; ** = p < 0.01; *** = p < 0.001.

An analysis was also performed with the FAs clustered by their number of unsaturation into monounsaturated FAs (MUFAs), PUFAs and saturated FAs (Fig 7). Supplementation with LA did not increase the total FA content of the reconstructed tissues (Fig 7A). However, interestingly, the proportions of each class of fatty acids were modified in the supplemented tissues (Fig 7B and 7C). The proportion of saturated FAs was higher in the reconstructed adipose tissues than in native fat (47 ± 3% and 30 ± 2% respectively; p < 0.001) (Fig 7B). This proportion was reduced when reconstructed tissues were supplemented with LA (40 ± 3%; p < 0.01) (Fig 7B). However, the proportion of saturated FAs in the phospholipid fraction was similar between samples (p > 0.05; Fig 7C).

We further investigated the impact of LA supplementation by performing a combined supplementation with a specific Δ-6-desaturase inhibitor (SC-26196, 100 mM, IC50). This addition partially blocked the increase in the proportion of LA in cells when supplemented with ω6 FAs (S1 Fig). Phase contrast microscopy during the cell differentiation process allowed to observe that supplementation with LA changes the morphology of differentiating adipocytes: instead of clusters of highly differentiated adipocytes (S1B, S1F and S1J Fig), differentiated cells seem to be distributed more evenly across the cell sheet (S1C, S1G and S1K Fig). Addition of the Δ-6-desaturase and LA reduce cell differentiation (S1D, S1H and S1L Fig) while addition of the Δ-6-desaturase alone had no notable effect on cell sheet development and maturation (S1E, S1I and S1M Fig). Vehicles (ethanol and DMSO) had no detectable impact on the production of the cell sheets (data not shown) nor on lipid profiles (S2 Fig) at the concentrations used in the experiments.

Robust MANOVA bootstrapped statistical analysis revealed that there was a significant main effect of the culture conditions (LA, inhibitor) on the levels of the 42 FAs measured (F = 4.32, P = 1.43 x 10^−6^) meaning that it was the parameter with the highest impact on lipid profile.

### Effect of LA supplementation on the eicosanoid and TR/RXR pathways

A physiologically relevant pharmacological model of adipose tissue needs to be able to produce inflammation related mediators such as prostaglandins, leukotrienes and thromboxanes. We used the Ingenuity’s molecule activation predictor (MAP) tool to visualize the effect of gene expression variation on the eicosanoid pathway (p = 7.07 x 10^−5^, S3 Fig), which was shown to be enriched with DEGs. The MAP tool of IPA predicted the overall effect of these DEGs on the pathways, based on their respective fold-change. In hrAT, the reduced expression of PLA2 predicts a decrease in arachidonic acid availability. Indeed, the enzymes involved in the lipid metabolism pathway, like cyclooxygenases (COX) and lipoxygenases (LOX), use ω6 family of PUFAs as their principal substrates. PUFAs are liberated from the phospholipidic membrane by the phospholipase A2 (PLA2) and are processed into molecules of the eicosanoids pathway. Arachidonic acid metabolism shows dysregulation toward synthesis of proinflammatory metabolites downstream of prostaglandin H2 (PGF2a, PGD2, PGI2, PGE2) and reduction of leukotrienes (B4, D4 and E4). Lower availability of arachidonic acid in hrAT seems to affect phospholipase A2 expression (downregulated). Consequently, leukotrienes B4, D4 and E4 are predicted to be reduced. On the other hand, a decrease of arachidonic acid would lead to an increase of prostaglandin H2. Thus, a decrease of thromboxane A2 is predicted as well as an increase expression of several prostaglandins. The measured expression of the downstream receptors corroborates the predictions for these prostaglandins except for three of them (*PTGER3*, *PTGER4*, *PTGDR*). Those results are in agreement with the demonstration of a deficiency in PUFAs in the culture environment. Eicosanoids cannot be accumulated in the cell as their half-life is very short (4–60 sec). They are rather released from the plasma membrane when summoned in response to cytokines and hormones [47]. Major inflammatory conditions such as asthma, hypertension and cancer are associated with deregulation of the eicosanoid pathway [48]. Therefore, there is a need for a relevant *in vitro* adipose model of this paramount tissue to be used in drug development and fundamental research.

We also visualized the effect of gene expression variation on the thyroid hormone receptor/retinoid X receptor (TR/RXR) pathway because many DEGs were part of this important pathway (Fig 3 and S4 Fig). Among others, upregulation of inflammatory mediators such as nuclear factor (NF)-κB, interleukin (IL)-6, IL-1β, COX-2 and inducible nitric oxide synthase (iNOS) were observed. In opposition, genes related to lipogenesis were predicted to be downregulated (S4 Fig).

## Discussion

Human ASCs extracted from native adipose tissues were used to engineer reconstructed AT serving in our laboratory as models to study adipogenic differentiation, triglycerides accumulation and metabolic functions. In the present study, we carried out the first extensive analysis of these 3D hrAT using transcriptomic and analysis of fatty acids profiles. The physiological relevance of the model was first assessed through microarrays and Ingenuity pathway analyses that showed genes and pathways differentially expressed in hrAT compared to native adipose tissues from healthy donors. However, the enrichment of the fatty acid metabolism pathway observed in hrAT motivated the subsequent analysis of their lipid and phospholipid profiles. These analyses revealed a deficit in ω6 essential fatty acids in the *in vitro* culture condition compared to the adipose tissue of individual representative of the North American population. Therefore, we show that optimisation of the culture conditions, by addition of LA in the culture medium during the engineering of hrAT, modulates cell total lipid content and the membrane phospholipids profile. This is especially important for the detailed study of molecules impacting on lipid metabolism using in vitro models.

Analysis of fatty acids was performed and reported both as percentages of total lipid content and as percentages within the membrane phospholipids. This study allows us to confirm that it is possible to modulate fatty acid of the cell membrane phospholipids by adjusting the composition of the cell culture media used for the production and maintenance of in vitro 3D engineered tissues.

Transcriptome analysis using bioinformatics tools predicted the modulation of pathways within particular biological functions that are altered in reconstructed compared to native tissues. As expected, upregulation of vitamin C transport and glycolysis pathways reflect the supplementation of the culture medium with vitamin C (50 mg/L) and glucose (4.5 g/L). Biological functions related to lipid metabolism (fatty acid α-oxidation, triacylglycerol degradation and phospholipase C signaling) were predicted to decrease. This could be explained by a lower availability of fatty acids. The mean fatty acid level in the cell culture media used was 46 ± 49 μg/ml, while normal serum level of triglycerides is 30 times higher (about 1.5 mg/ml). Therefore, lipid supplementation of the culture media would allow restoring a more physiological level and allowing for standardization between batches. The high SD of the mean highlights the inconsistency between FCS batches and supports the use of defined lipid serum solutions.

Our results are in accordance with the effects of LA supplementation observed in studies using *in vitro* cultured rodent cells (3T3-L1) by Petersen *et al*. [20]. They demonstrated that both LA and arachidonic acid supplementation increased the expression and activation of PPARγ, resulting in TG accumulation. Brown *et al*. demonstrated hypertrophy of lipid droplets by evaluating the effect of supplementation with linoleic isomers on preadipocyte differentiation [49]. Madson et al. demonstrated that polyunsaturated fatty acids regulate adipocyte differentiation and function [19]. Moreover, when the lipid profiles of our donors were compared to other published studies on Canadians like those presented in Caron-Jobin et al. [45], we obtained similar FA profiles (42 FAs) except for the oleic acid that was slightly higher in our donors (15%).

The high content of glucose in the culture medium (4.5 g/L) is presumed to be responsible for the increase in saturated and monounsaturated fatty acids (palmitic, palmitoleic and oleic) since it is the direct product of lipogenesis in adipocytes. In vivo, de novo lipogenesis only account for approximately 20% of the fatty acids stored in adipocytes. Glucose level in normal human serum is between 0.83 to 1.15 g/L [50]. Saturated fatty acids are recognized to have deleterious effects on the cardiovascular system [51–53]. By modulating the level of glucose and FAs in the culture medium, it would be possible to reproduce *in vitro* situations representative of healthy and unhealthy diets. The capacity of our engineered adipose tissue model to adapt to the *in vitro* culture environment could be useful in the study of dysregulation and pathophysiological processes such as obesity, metabolic syndrome, dyslipidemia and diabetes. A pathological model of engineered adipose tissue could contribute to the identification of new therapeutic drug targets in area of intervention such as lipid management and inflammation. Engineering of adipose tissues has been achieved using different biomaterials including synthetic polymers, [54, 55], collagen microbeads [56], decellularized matrix [57] and collagen sponges (collagen, fibronectin) [58, 59]. The self-assembly approach we used in this study has the advantage of being composed of cell-produced extracellular matrix procuring a more native-like microenvironment. The latter would therefore be highly relevant to study integrated pathological processes affecting both the cells, their lipid contents and the matricial elements of the adipose tissue depots.

The cell-membrane phospholipid composition is variable between cell types and is directly related to their function. Hence, similar studies could be conducted in tissue-engineered models of other relevant tissues to ensure that the appropriate fatty acids are provided. For example, tissue-engineered skin models [60–62] could benefit from LA supplementation since the deficit in essential FAs is recognized to reduce water retention in the epidermis by affecting ceramide composition [63].

One limitation of the comparison between native and reconstructed tissue is the presence of other cell types than adipocytes in the native fat used. As a future perspective, it would be interesting to develop a rapid separation technique that would not affect gene expression in order to perform single cell transcriptomic analysis. Therefore, we have to consider that the native tissue comprises heterogeneous cell populations that could explain the wide distance between native and reconstructed tissues in the PCA analysis (Fig 2).” However, to limit the impact of this heterogeneity in cell types, we focused the analyses on pathways related to lipid metabolism, as fatty acids are the building blocks for phospholipids and triglycerides in differentiated adipocytes.

In conclusion, this study highlights that the lack of essential fatty acids in the culture medium should be addressed, in particular for adipose tissue engineering applications. We demonstrated that native adipose tissues and our hrAT display 8% of difference in their transcriptome. Differences in the eicosanoids pathway as well as saturated and essential FA profiles were observed. The analysis of the fatty acid profile pattern revealed that essential FAs are beneficial for the engineering of a more relevant *in vitro* model of human adipose tissue since LA supplementation re-established the fatty acid profile. Therefore, these studies will contribute to a better comprehension of adipose tissue formation and of fatty acid metabolism as well as being a promising alternative for soft tissue reconstruction.

## Supporting information

S1 Fig Effect of LA supplementation and Δ-6 desaturase inhibition on lipid accumulation. (A) Analysis of the lipid content by gas chromatography of hrAT supplemented with linoleic acid (LA). The delta-6 desaturase inhibitor (SC-26196) was added at the IC50 (100 mM) to avoid cytotoxicity. Vehicles were added to the controls (n = 3). Phase-contrast microscopy images of adipose cell sheets after 3 (B-E), 13 (F-I) and 18 (J-M) days of supplementation for control hrAT (B, F, J); LA 150 μM (C, G, K); LA 150 μM + SC-26196 100 mM (D, H, L); SC-26196 (100 mM; E, I, M), Scale bar = 200 μm.
(TIF)

S2 Fig Effect of solvents on the lipid profile of engineered adipose tissues. Gas chromatography analysis of the lipid content of reconstructed adipose sheets treated with vehicles ethanol 0.1% and/or DMSO 0.5% V/V.
(TIF)

S3 Fig Variation between engineered and native adipose tissues for the eicosanoids signaling pathway. Transcripts colored in blue and orange are downregulated and upregulated, respectively. DEGs with a p<0.05 and a fold change > 2 were included into Molecule Activity Predictor (MAP).
(TIF)

S4 Fig Variation between engineered and native adipose tissues in relation with the TR/RXR pathway. Transcripts colored in blue are downregulated while those appearing in orange are upregulated. DEGs with a p<0.05 and a fold change > 2 were included into Molecule Activity Predictor (MAP).
(TIF)
