# Supplementary figures and images for: Linoleic acid supplementation of cell culture media influences the phospholipid and lipid profiles of human reconstructed adipose tissue
Source: PLoS One. 2019 Oct 22;14(10):e0224228. doi: 10.1371/journal.pone.0224228 (PMC6805161; doi:10.1371/journal.pone.0224228)

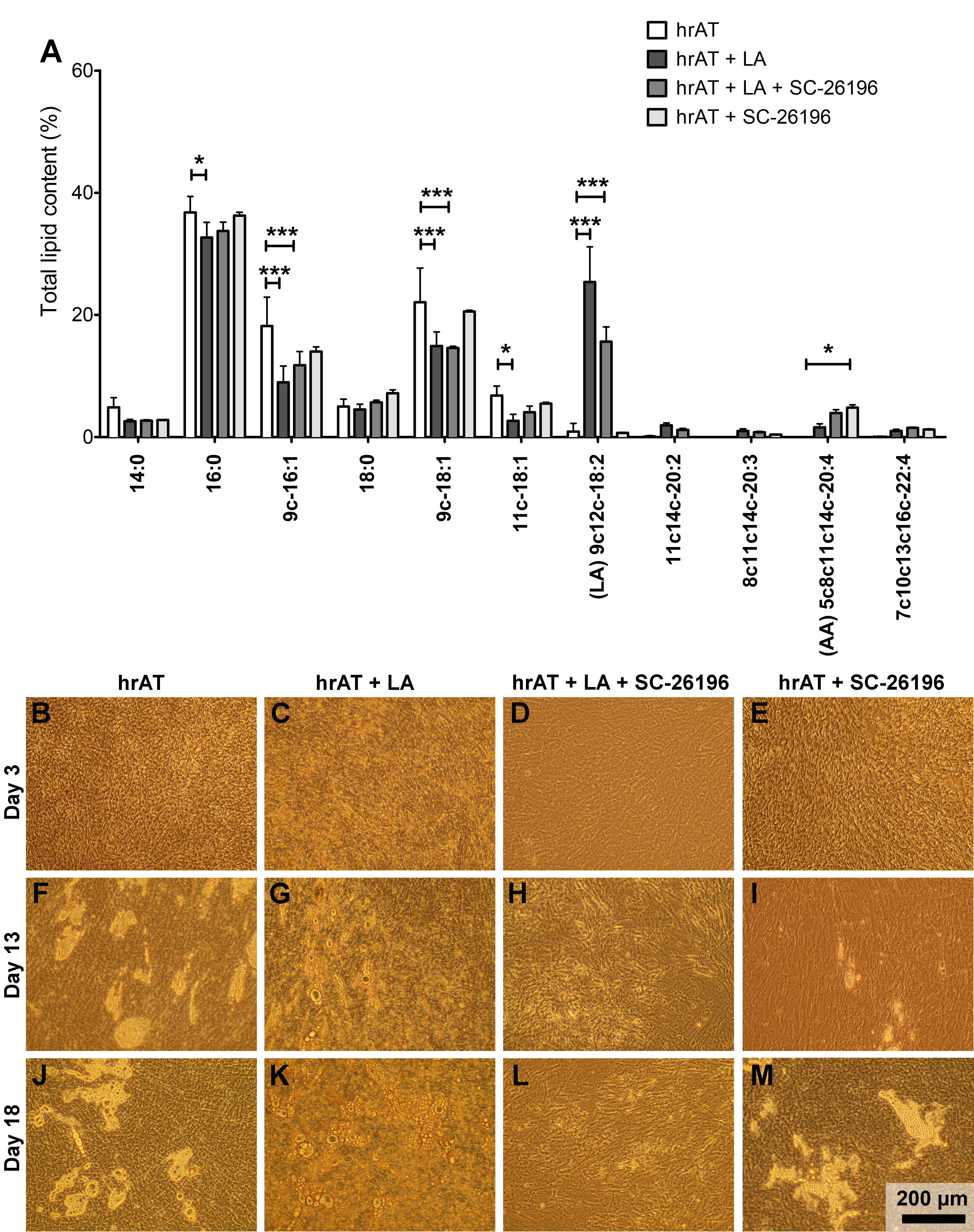

Supplement: S1 Fig — (A) Analysis of the lipid content by gas chromatography of hrAT supplemented with linoleic acid (LA). The delta-6 desaturase inhibitor (SC-26196) was added at the IC50 (100 mM) to avoid cytotoxicity. Vehicles were added to the controls (n = 3). Phase-contrast microscopy images of adipose cell sheets after 3 (B-E), 13 (F-I) and 18 (J-M) days of supplementation for control hrAT (B, F, J); LA 150 μM (C, G, K); LA 150 μM + SC-26196 100 mM (D, H, L); SC-26196 (100 mM; E, I, M), Scale bar = 200 μm. (TIF) [file pone.0224228.s001.tif]

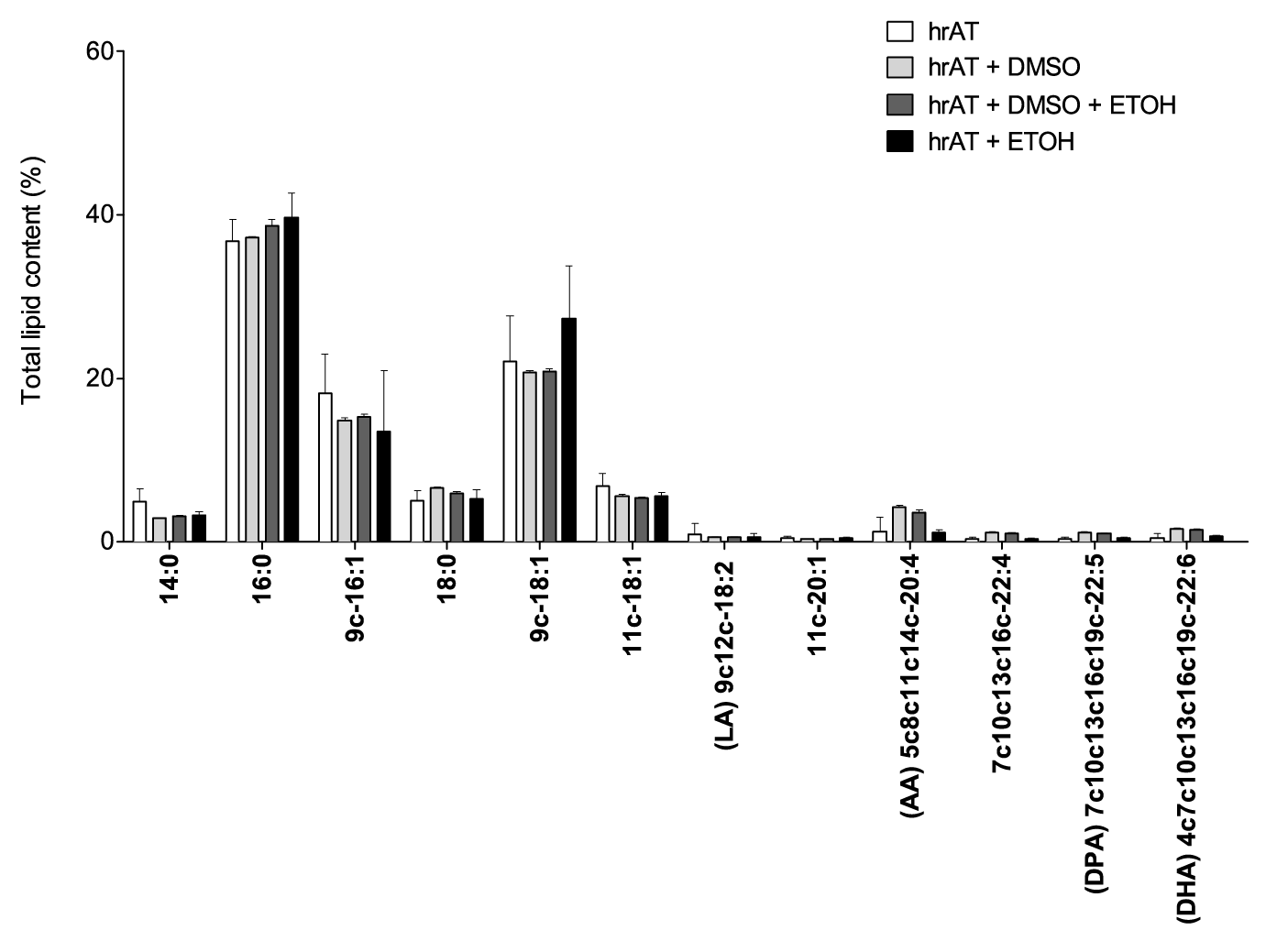

Supplement: S2 Fig — Gas chromatography analysis of the lipid content of reconstructed adipose sheets treated with vehicles ethanol 0.1% and/or DMSO 0.5% V/V. (TIF) [file pone.0224228.s002.tif]

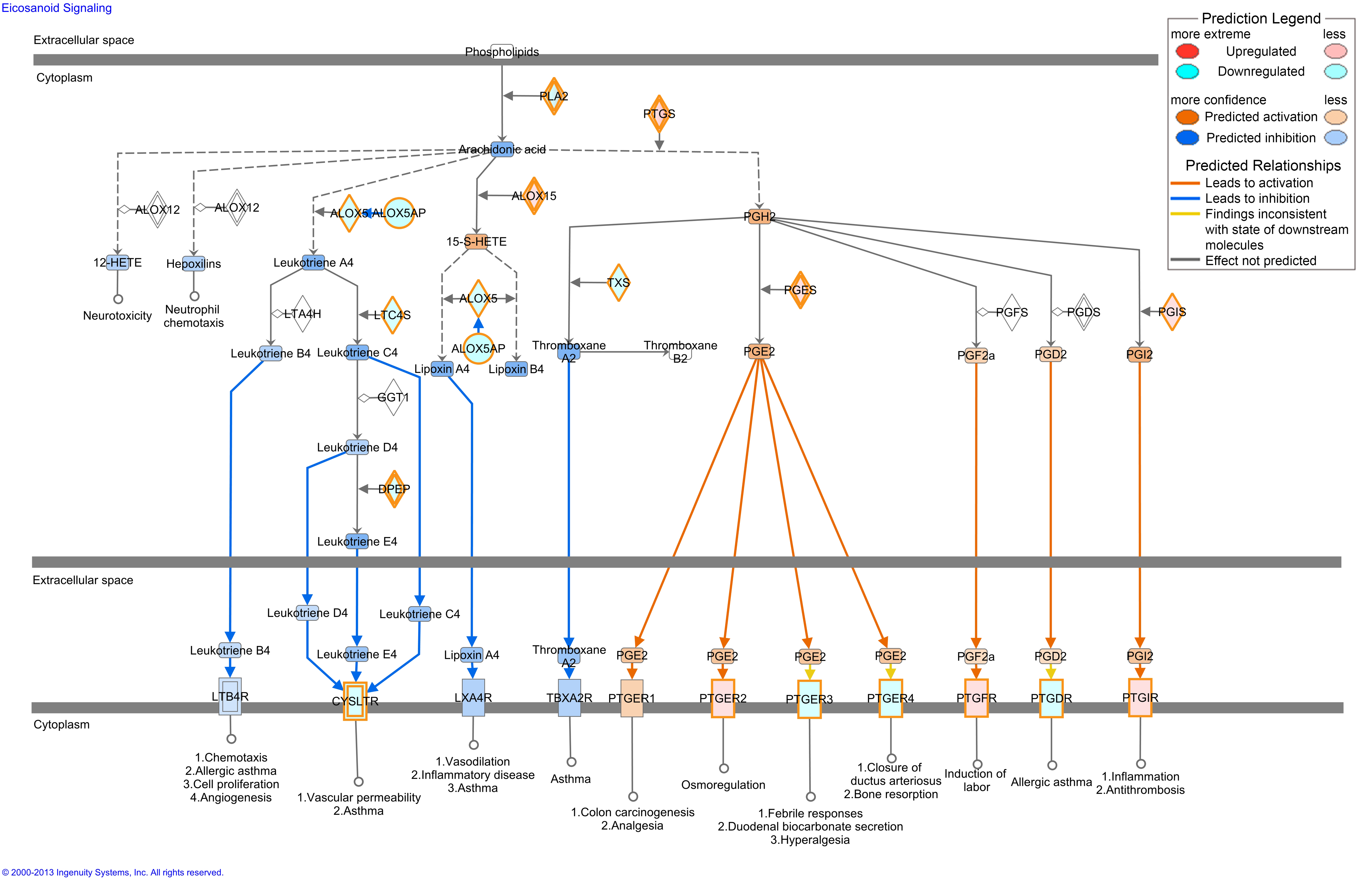

Supplement: S3 Fig — Transcripts colored in blue and orange are downregulated and upregulated, respectively. DEGs with a p<0.05 and a fold change > 2 were included into Molecule Activity Predictor (MAP). (TIF) [file pone.0224228.s003.tif]

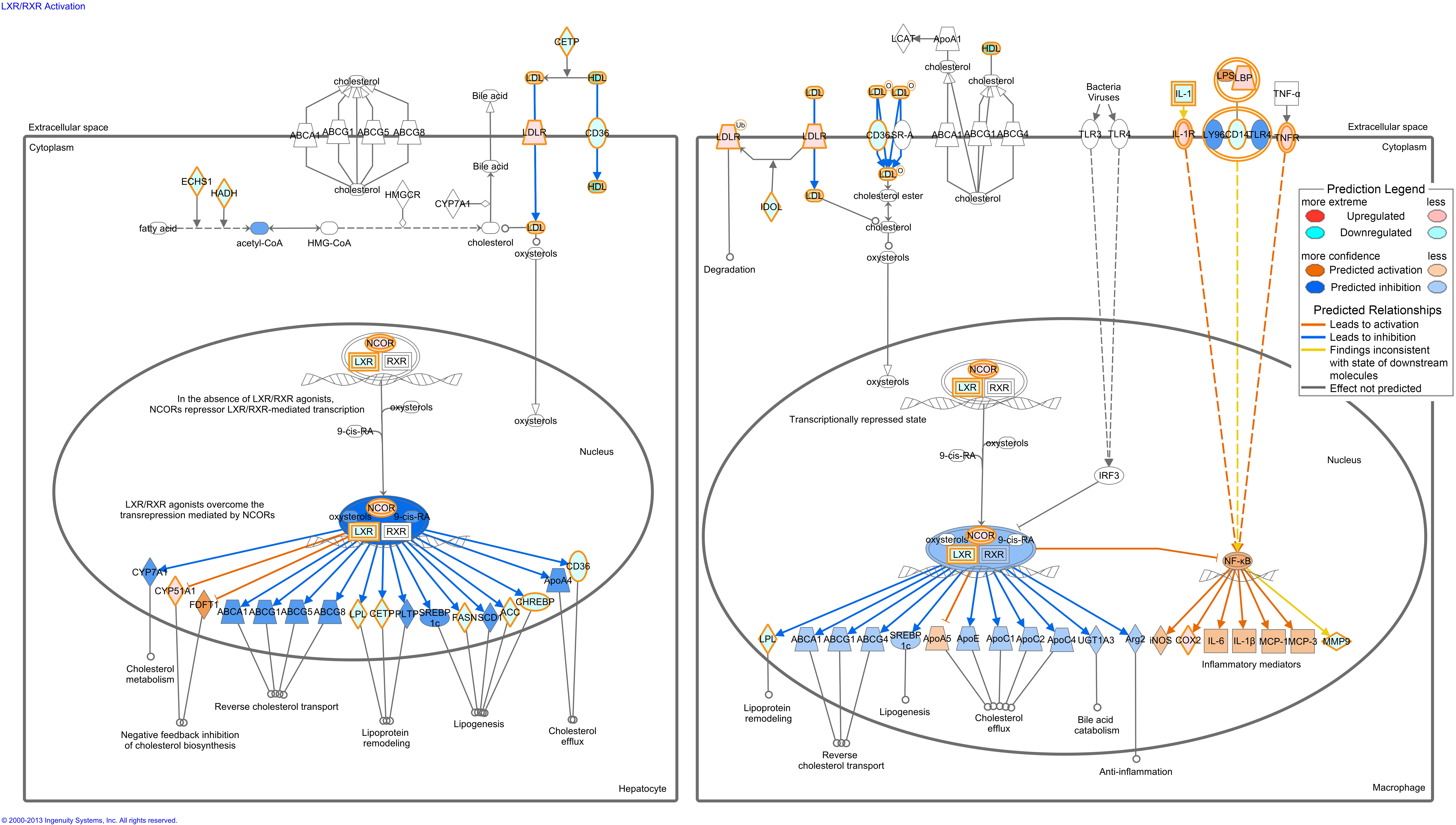

Supplement: S4 Fig — Transcripts colored in blue are downregulated while those appearing in orange are upregulated. DEGs with a p<0.05 and a fold change > 2 were included into Molecule Activity Predictor (MAP). (TIF) [file pone.0224228.s004.tif]
